# Molar Incisor Hypomineralisation—To Extract or to Restore beyond the Optimal Age?

**DOI:** 10.3390/children7080091

**Published:** 2020-08-06

**Authors:** Mustafa Elhussein, Hasan Jamal

**Affiliations:** 1 Faculty of Dentistry, Ibn Sina University, Khartoum P.O.Box 10995, Sudan; mustafa_elhussein@hotmail.com; 2 Eastman Dental Institute, University College London, London WC1X 8LD, UK

**Keywords:** paediatric dentistry, orthodontics, enamel defects, first permanent molars (FPMs), molar incisor hypomineralisation (MIH), post-eruptive breakdown (PEB), temporary anchorage devices (TADs)

## Abstract

The management of compromised first permanent molars (FPMs) in children presents a clinical challenge to the dental team. Hypomineralised FPMs in molar incisor hypomineralisation (MIH) conditions could undergo post-eruptive breakdown, making them susceptible to caries, leading to their subsequent loss. The planned extraction of compromised FPMs is a valid alternative to complex restorative treatment. However, establishing the presence or absence of third permanent molars, amongst other considerations, is crucial to reaching a successful outcome. Clinicians should understand the importance of an orthodontic examination around the age of 8 years old with regard to establishing a differential therapeutic decision about the ideal timing of MIH-affected FPMs’ extraction in children. The aim of this article is to highlight that, with an interdisciplinary approach, a good outcome can be achieved following the extraction of poorly prognosed FPMs. The most cost-effective way of addressing MIH-affected FPMs is extraction, followed by orthodontic space closure when indicated. This obviates the need for the repeated restorative replacement and saves perfectly healthy premolars from being extracted for space creation in orthodontic treatment in several clinical scenarios.

## 1. Introduction

The term molar incisor hypomineralisation (MIH) was first introduced in 2001, and defined as ‘demarcated, qualitative developmental defects of systemic origin of the enamel of one or more first permanent molar (FPM) with or without the affection of incisors’ [1]. More recently, new patterns have been observed, such as the cusp tips of permanent canines and premolars. hypomineralised second primary molars (HSPMs), which can also affect the cusp tips of permanent canines and premolars [2,3,4]. It was also noted that, when there are HSPMs, there is a 50% chance that the FPMs may also be affected [5].

### 1.1. Aetiology

Although the aetiology of MIH remain unclear, several causes have been hypothesised. They could be grouped into either maternal or neonatal. For the maternal causes, it could be a nutritional, infectious, haematological, metabolic or endocrinal disturbance. The neonatal causes could be related to premature birth, systemic upset, nephrotic or gastrointestinal disturbances. Recent studies have also shown a possible genetic link to MIH [6,7].

### 1.2. Prevalence

A recent systematic review reported that, globally, MIH affects 878 million individuals, with an annual prevalence of 17 million [8]. Other regional studies have shown that MIH’s prevalence ranges from 2.4% to 40.2% in studies conducted among populations in Hong Kong, Denmark and Brazil [9,10,11]. The wide variations in regard to prevalence are thought to be due to a lack of uniformity in the use of classification indexes, which were utilised inconsistently and with no standardisation.

### 1.3. Classification

MIH lesions are characterised by being well-demarcated opacities. The colour, the extent of the lesion and the level of sensitivity differ depending on the severity of the condition. Numerous classification criteria were developed to classify MIH lesions. The European Academy of Paediatric dentistry first classified MIH in 2003. The key features for this classification are the demarcation of the opacity, enamel disintegration, atypical restorations, sensitivity, extracted and unerupted teeth [12].

A more basic classification has also been suggested for MIH, which could either be mild or severe [13]. In mild conditions, the demarcated opacities are not associated with a post-eruptive breakdown (PEB), with occasional sensitivity to external stimulus and with less aesthetic concerns. While in severe cases, the demarcated enamel is associated with PEB, spontaneous sensitivity and high aesthetic demands.

Another classification was developed, which classified MIH into three categories, mild, moderate or severe [14]:

1. Mild MIH: The demarcated opacities located at non-stress bearing areas, no caries associated with the affected enamel, no hypersensitivity and incisor involvement is usually mild if present;
2. Moderate MIH: The demarcated opacities present on molars and incisors, the post-eruptive enamel breakdown limited to one or two surfaces without cuspal involvement, atypical restorations can be needed and normal dental sensitivity;
3. Severe MIH: Post-eruptive enamel breakdown, crown destruction, caries associated with affected enamel, a history of dental sensitivity and aesthetic concerns.

A detailed scoring system was then put forward, which quantifies MIH’s severity according to the number of teeth affected along with the type and extent of the defect [15]. More recently, a more subjective index was proposed, referred to as MIH treatment need index (MIH-TNI). This index was based on describing the population’s treatment needs, and it is based on two factors, hypersensitivity and PEB [16].

### 1.4. Clinical Considerations

Current research demonstrated that children presenting with MIH (moderate to severe) usually require life-long, extensive and repeated restorative treatments that will eventually fail [17]. Studies have shown that children with MIH exhibited 11 times greater probability of having their teeth restored compared to unaffected children. It has been demonstrated that fissure sealants and fillings used in affected FPMs had over three times greater probability of requiring re-treatment compared to children with unaffected FPMs; this is also complicated with anxious children [18,19].

These failures are primarily related to the mechanical, structural and compositional defects of MIH-affected teeth. Under scanning electron microscopy (SEM), the enamel prisms were shown to have less dense and with less organised hydroxyapatite crystals [20,21]. Regarding composition, transmission electron microscopy (TEM) data have shown that MIH-affected teeth have reduced mineral density and with significantly high protein content [22], in comparison to sound enamel [23,24]. Concerning the mechanical properties, the main aspect of being assessed is the hardness and the modulus of elasticity. MIH-affected teeth were shown to have significantly reduced hardness and elasticity levels in comparison to sound enamel. Several clinical and laboratory studies compared MIH-affected molars and molars with sound enamel concerning dental composite bonding strength to enamel substrate. They have reported that the bond strength to MIH-affected enamel was significantly less in comparison to molars with sound enamel [25]. Regarding the cavity design, studies have shown that if the cavity designs were invasive (i.e., removal of all affected tooth structure), the success rate would be higher in comparison to non-invasive design [26]. Nonetheless, the compromise of sound enamel will result in further tooth weakening, and ultimately, this will compromise the longevity of composite fillings. As highlighted previously, MIH-affected teeth have increased protein content, which directly affects the bond strength. Thus, deproteinization was suggested as a method to enhance bond strength. Although some studies showed improvement in bond strength following deproteinization, there is weak evidence to prove this [27]. This, in return, will negatively affect the restoration-support by the tooth surface [21]. There is also emerging and growing evidence of the influence of MIH on children’s oral health-related quality of life in terms of aesthetic concerns related to the maxillary incisors [28,29,30].

### 1.5. Management Strategies: Cost Effectiveness and Long-Term Prognosis

Management of moderate to severely affected FPMs can be summed up into three categories; direct restorations (e.g., composite); indirect restorations (e.g., gold-onlays); or extraction (aiming for spontaneous or orthodontic intervention to close the gap). However, due to the aforementioned compositional and structural defects of severely affected FPMs, the restorative option could be questionable and more expensive in most of the cases. A recent model-based analysis aimed to analyse the cost effectiveness of different treatment options for severely affected FPMs [31]. The treatment options included in the analysis were extraction (with or without orthodontic space closure), composite restorations or indirect restorations. Based on their findings, they concluded that the extraction of severely affected FPMs followed by orthodontic alignment was the most cost-effective option with a good long-term prognosis compared to the restorative option [31].

### 1.6. The Orthodontic Interface: Timely Versus Delayed Extractions

It is crucial for general dental practitioners, paediatric dentists, and orthodontists to adopt appropriate clinical guidelines [32,33,34,35]. Guidelines on treatment planning FPMs’ extraction in children within the context of malocclusion have been produced. The Royal College guidelines is considered to be the most often cited aid decision-making tool in the UK [35]. However, even when these guidelines are adopted, the management of compromised FPMs yield significant amounts of confusion, which recent articles aimed to clarify [36,37].

The most cost-effective way of addressing the loss of FPMs is orthodontic space closure because it obviates the need for restorative-prosthetic replacement and saves dentally sound premolars from being extracted for space creation in orthodontic treatment. Therefore, a meticulous treatment planning process and an interdisciplinary approach, which depends upon excellent lines of communication between the general dental practitioner, or paediatric dentist, and orthodontist, are essential for achieving excellence in patient care [38].

The extraction of FPMs is considered to be technically more difficult; even a good result is in some way a compromise [39]. Many cases that would benefit from this approach because of the questionable long-term prognosis of these molars are treated with the extraction of perfectly sound premolars. Sandler et al. [39,40] highlighted several reasons for the avoidance of FPMs extraction cases:

▪ Clinician comfort with orthodontics cases involving the planned extraction of premolars for space creation;
▪ Lack of experience in handling FPM extraction cases;
▪ Presence of an inter-dependence for patients between dental specialists in some financially driven healthcare systems.

Nevertheless, the planned extraction of FPMs, is dependent on certain circumstances, including, but not limited to [39]:

- Level of oral hygiene;
- Patient and parent motivation to orthodontic treatment;
- Amount and site of crowding;
- Presence or absence of other permanent teeth, particularly the third molars.

It is of paramount importance therefore to consider these circumstances to ensure a patient’s suitability for such invariably lengthy course of fixed appliance therapy. Depending on the patient’s age, the decision to extract FPMs would ideally be made by an interdisciplinary team, which includes the clinician who is involved in patient’s dental care, and the orthodontist who will ultimately be providing the fixed appliance therapy.

## 2. Cases Presentation

### 2.1. Case 1—Delayed Extraction of FPMs

**Figure 1:**
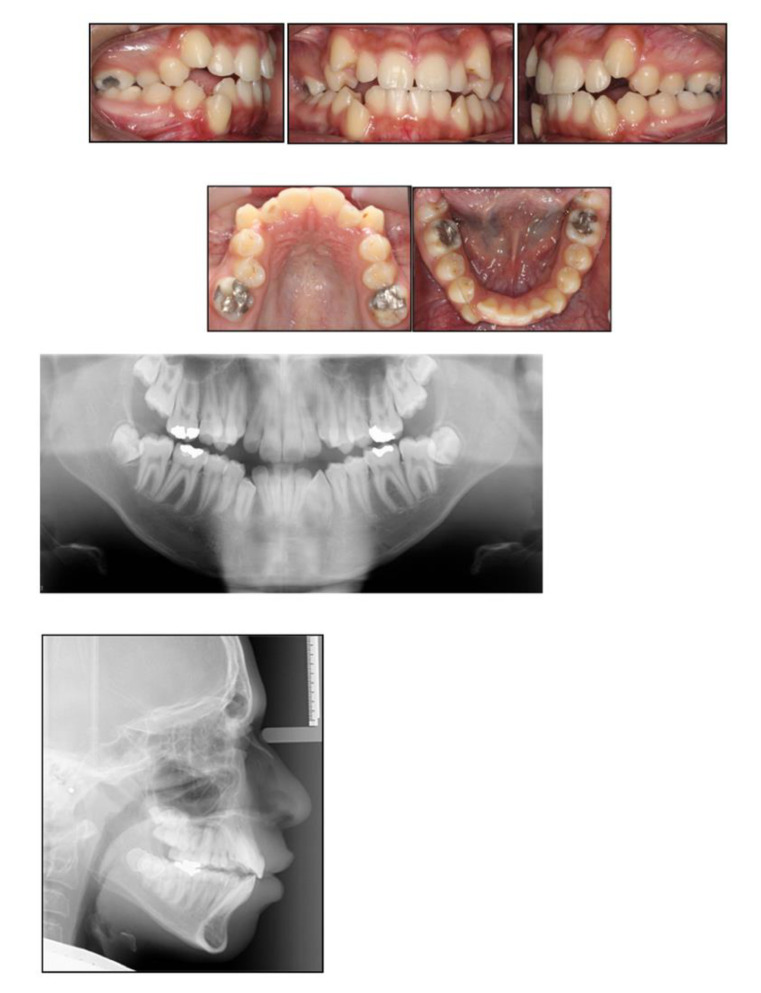
Pre-treatment intraoral photographs and radiographs.

A 15-year-old male, presented with a Class I incisor relationship on a mild Class II skeletal base with increased vertical proportions (Figure 1). This was complicated by heavily restored upper first permanent molars, restored lower first permanent molars, reduced overbite, moderate upper arch and severe lower arch crowding, and a lower centreline discrepancy to the right side (Figure 1). The maxillary central incisors showed white opacities. His dental history revealed repeated and major restorative work associated with his MIH-affected FPMs. The patient also presented with signs and symptoms associated with the heavily restored upper FPMs. His Orthopantomograph (OPT) showed signs of molar stacking of the upper right and left second molars, and distally tipped lower right and left second molars (Figure 1). His paediatric dentist referred him to explore the potential for fixed appliance therapy and, if indicated, whether or not extractions of FPMs will be planned as part of orthodontic treatment.

**Figure 2:**
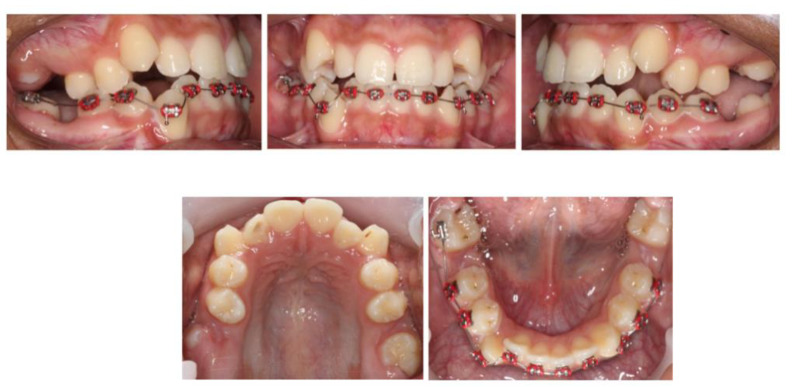
Six months following the extraction of all first permanent molars (FPMs). Note the favorable spontaneous eruption of the second permanent molars. The placement of full fixed appliances was postponed until complete eruption of the second permanent molars.

With a view to help the patient avoid a course of root canal treatment to his upper FPMs, treatment involved the extraction of the upper and lower first permanent molars, together with upper and lower fixed appliances (Figure 2).

#### Discussion

Although the timing of FPMs’ extraction is most favourable around a chronological age of 8–10 years old, this older case illustrates the advantages of FPMs’ extraction at a later stage. The extraction pattern in this case was dictated primarily because of the pain, large restorations and the questionable long-term prognosis of the upper FPMs, and because of the large occlusal restorations on the lower FPMs. Furthermore, extraction in the lower arch was indicated, because of the severity of the crowding and space requirements.

In this case, the extraction of FPMs increased the prognosis of the remaining dentition and relieved the signs and symptoms associated with upper FPMs. If the FPMs had not been of poor prognosis, the extraction pattern might have been the extraction of the first premolars. Although the response of the second permanent molars is variable following extraction of FPMs and acceptable positions would also be achieved irrespective of the timing of extraction [40], a favourable mesial eruptive position of second permanent molars was predictable, to some extent, in this case due to a number of reasons:

▪ The upper second permanent molars were still within bone at the time of extraction;
▪ The OPT revealed signs of molar stacking of upper right and left second permanent molars, and distally tipped lower right and left second permanent molars reflecting posterior molar crowding;
▪ The distinct vertical growth pattern and presence of a steep mandibular plane, which encouraged molar mesial movement.

**Figure 3:**
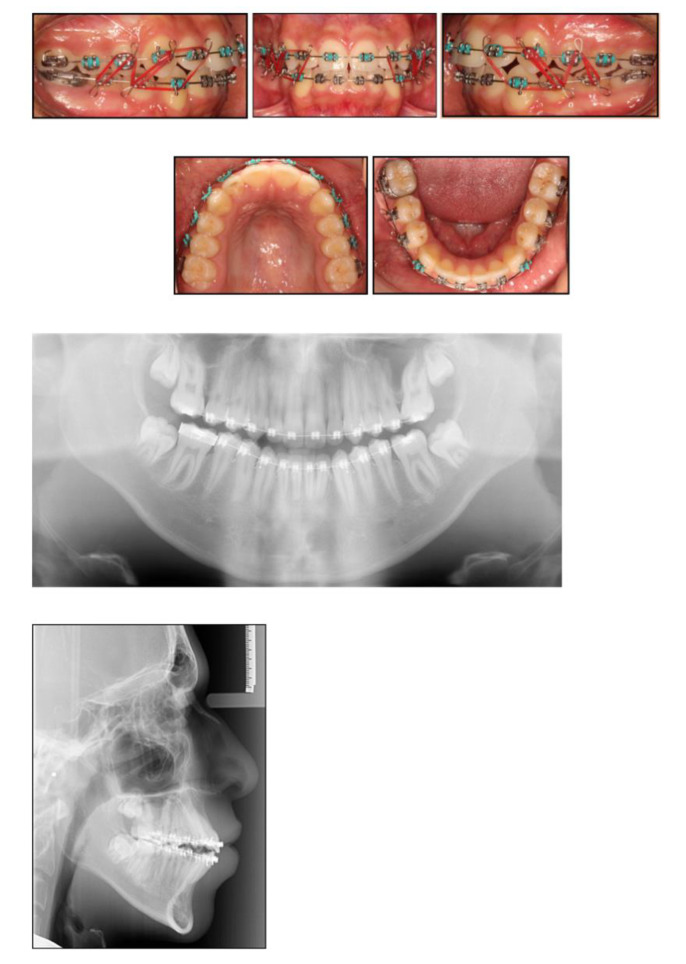
Near-end-of-treatment stage following space closure. Near-end-of-treatment radiographs revealed favourable third molar positioning. Intermaxillary elastics with a vertical vector were utilised to actively enhance interdigitation.

**Figure 4:**
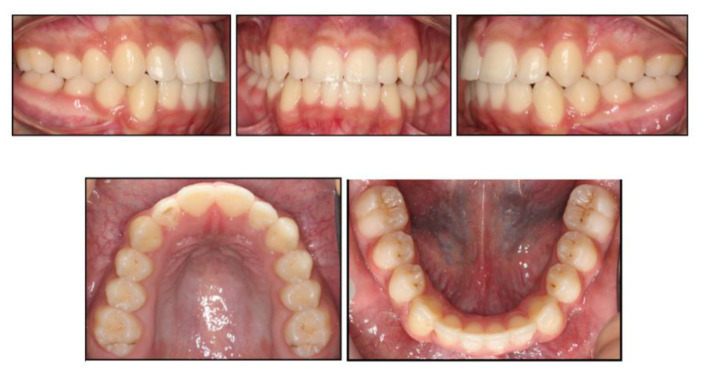
End-of-treatment intraoral photographs. Space closure was fully achieved and orthodontic treatment was completed in 16 months.

Moreover, the OPT showed favourable positioning of third molars following space closure (Figure 3) Perhaps, in this case, if elective extraction of FPMs was prescribed around the optimum time, provided the third molars were radiographically detectable, the repeated restorative work might have been avoided. On the other hand, this approach could still be deemed cost effective, as it obviated the need for further dental treatment i.e., root canal treatment (Figure 4).

### 2.2. Case 2—Timely Extraction of FPMs

**Figure 5:**
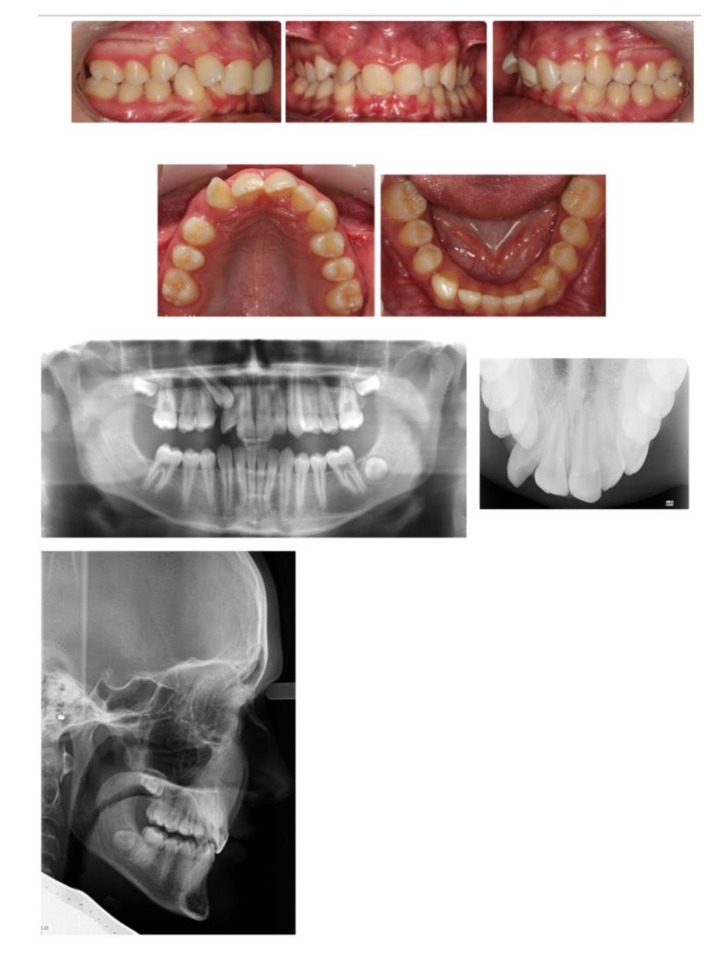
Pre-treatment intraoral photographs and radiographs. The extraction of all molar incisor hypomineralisation (MIH)-affected FPMs was carried out at the age of 9. Note the congenitally absent LR8.

A 16-year-old male, presented with a Class II division 2 incisor relationship on a mild Class II skeletal base with average vertical proportions. This was complicated by a moderately crowded upper arch, buccally impacted UR3, and increased overbite, which was complete to soft tissue with no evidence of trauma (Figure 5). The maxillary central incisors showed white and cream opacities. His dental history revealed delayed dental development, and also reported that his-MIH-affected FPMs were extracted at the age of 9. No orthodontic consultation had been sought at the time. His OPT moreover confirmed the presence of all third molars, apart from the lower right third molar. The upper standard occlusal radiograph showed no evidence of pathology associated with the upper right lateral incisor and upper right canine.

#### Discussion

The FPMs in this case report were extracted around the recommended optimal age (8–11), which led to successful spontaneous eruption of the second permanent molars (Figure 5). The space requirements in the upper arch, however, were deemed to be significant in this case; perhaps the temporization or restoration of the upper FPMs might have been a valid treatment alternative at the time. Additionally, the early extraction of the FPMs led to compromising the molar dentition in this case, due to the congenital absence of the lower right third molar (Figure 5). It is important to take into consideration the third molar’s presence when treatment planning for the extraction of FPMs, to ensure the remaining molars erupt in a position that maintains good functional occlusion.

In contrast to Class I cases, the extraction of FPMs in growing Class II cases is more critical to plan, particularly with regard to the timing of upper FPMs extraction. This is due to the space requirements often needed in the upper arch for the correction of the incisor relationship and increased overjet. Whilst a favourable mesial eruptive position of second permanent molars was achieved in this case, the timely extraction predisposed the patient to a number of malocclusion features, including (Figure 5):

▪ Round tipping of upper and lower second permanent molars;
▪ Distal tipping of lower second premolars;
▪ Spacing in the lower arch;
▪ Excessive retroclination of the lower labial segment that worsened an inherent deepbite.

The aforementioned features are a relatively common sequelae of the early extraction of FPMs in some Class II malocclusions, particularly the division 2 type, and they often present with a varying degree of severity.

## 3. Conclusions

Treatment planning for the extraction of FPMs can present a challenge, particularly in the presence of an underlying malocclusion. The available evidence for managing and prescribing FPMs extraction is considered weak, merely reflecting clinical opinion [35,37]. Up until today, there are no randomised prospective controlled trials reporting on the outcome of different interventions, hence yielding high-quality evidence.

With the presence of an orthodontist’s support, the extraction of FPMs should be encouraged. This valid treatment approach is not only cost effective, but it limits the repeated restorative events a child is normally subjected to, thus leading to increased anxiety in these children. It is worth mentioning that, orthodontic cases involving the extraction of FPMs has shown a 90% success rate of third molars’ eruption, compared to approximately a 55% chance with cases involving premolar extractions, therefore reducing future common complications associated with unerupted and impacted third molars [41,42].

**Figure 6:**
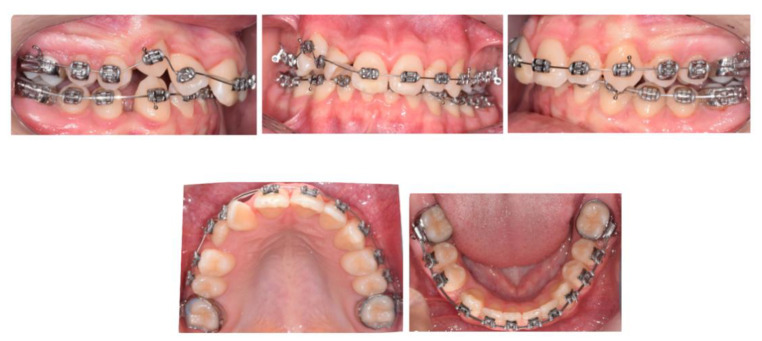
Fixed appliances and headgear were used to create sufficient space in the upper arch and align the teeth.

**Figure 7:**
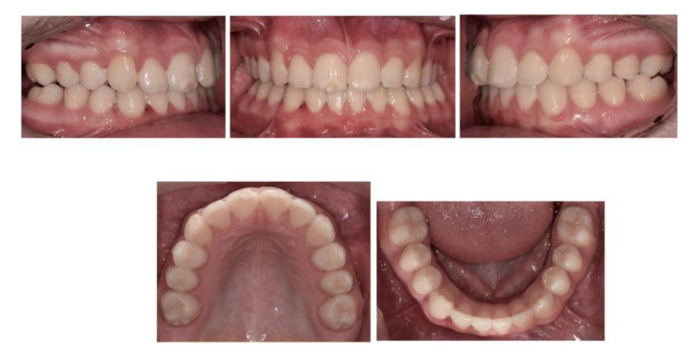
End-of-treatment intraoral photographs. Alignment, overbite, and overjet correction was achieved with fixed appliances in 18 months.

**Figure 8:**
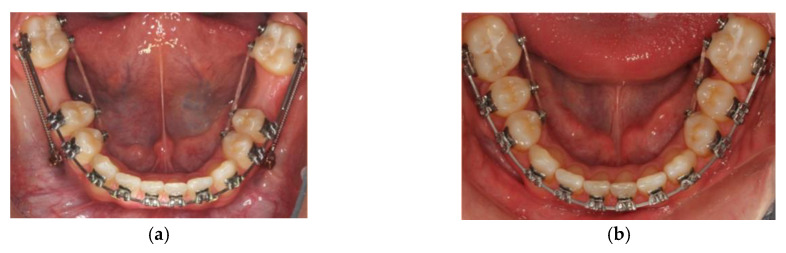
(**a**) Temporary anchorage devices were used to facilitate the space closure of 12 mm of space resulting from the planned extraction of the lower FPMs; (**b**) Spaces were closed after a period of 9 months.

An orthodontic examination around the age of 8 years old thus makes sense with regard to establishing a differential therapeutic decision, with regard to, the ideal timing of MIH-affected molar extraction. As described and illustrated in this paper, contemporary fixed appliances (Figure 6 and Figure 7) can achieve good and predictable treatment outcomes following the planned extraction of FPMs irrespective of the chronological ages, particularly with the advent of temporary anchorage devices, which are deemed versatile, thus facilitating space closure in challenging orthodontic cases (Figure 8) [43,44].

The unfolding COVID-19 pandemic rendered practice guidelines being focused around limiting Aerosol Generating Procedures (AGPs). This influences the provision of dental services of paediatric patients [45]. For the current predicament, and in the post-pandemic era, the aerosols generated from the repeated restorative work on such teeth, i.e., MIH-affected FPMs, might not be in the best interest of patients and dental team alike. As highlighted above, a holistic management strategy should be utilised to avoid AGPs.
